# Th17 Pathway As a Target for Multipotent Stromal Cell Therapy in Dogs: Implications for Translational Research

**DOI:** 10.1371/journal.pone.0148568

**Published:** 2016-02-12

**Authors:** A. Kol, N. J. Walker, M. Nordstrom, D. L. Borjesson

**Affiliations:** Department of Pathology, Microbiology and Immunology, School of Veterinary Medicine, University of California, Davis, California, 95616, United States of America; Wake Forest Institute for Regenerative Medicine, UNITED STATES

## Abstract

Detrimental Th17 driven inflammatory and autoimmune disease such as Crohn’s disease, graft versus host disease and multiple sclerosis remain a significant cause of morbidity and mortality worldwide. Multipotent stromal/stem cell (MSC) inhibit Th17 polarization and activation in vitro and in rodent models. As such, MSC based therapeutic approaches are being investigated as novel therapeutic approaches to treat Th17 driven diseases in humans. The significance of naturally occurring diseases in dogs is increasingly recognized as a realistic platform to conduct pre-clinical testing of novel therapeutics. Full characterization of Th17 cells in dogs has not been completed. We have developed and validated a flow-cytometric method to detect Th17 cells in canine blood. We further demonstrate that Th17 and other IL17 producing cells are present in tissues of dogs with naturally occurring chronic inflammatory diseases. Finally, we have determined the kinetics of a canine specific Th17 polarization in vitro and demonstrate that canine MSC inhibit Th17 polarization in vitro, in a PGE_2_ independent mechanism. Our findings provide fundamental research tools and suggest that naturally occurring diseases in dogs, such as inflammatory bowel disease, may be harnessed to translate novel MSC based therapeutic strategies that target the Th17 pathway.

## Introduction

T_help_17 (Th17) driven inflammatory and autoimmune diseases such as multiple sclerosis, Crohn’s disease, psoriasis, rheumatoid arthritis and graft versus host disease remain a significant source of morbidity and mortality worldwide.[1–5] Th17 cells are a subset of T helper cells (i.e. CD4+ T cells) that are defined by their capacity to secrete IL17 family member cytokines (IL17A-E) upon activation.[6–9] IL17 family members are potent pro-inflammatory cytokines that induce the production and secretion of numerous other pro-inflammatory cytokines, chemokines, hematopoietic growth factors and prostaglandins by neighboring epithelial, endothelial and stromal cells.[6] In turn, these factors lead to fever, systemic inflammation, increased granulopoiesis and the recruitment of neutrophils, macrophages and activated T cells.[6] IL17 cytokines are also secreted by non-Th17 cells including CD8+ T cells (aka Tc17), γδ-T cells and innate lymphoid cells and their roles in homeostasis and disease are just beginning to be explored.[10–13]

There is an urgent and unmet need to increase the number of US Federal Drug Administration (FDA) approved novel therapeutics to target Th17 mediated diseases.[14,15] These disorders result from complex interactions between the patient’s genetic and epigenetic background and environmental effectors,[16–18] interactions that are poorly mimicked by traditional induced-models-of-disease in rodents.[19,20] Increasingly, the translational relevance of naturally occurring diseases in companion animals is being explored to bridge the gap between clinical trials in human beings and rodent models of disease.[19–21] Naturally occurring idiopathic inflammatory and autoimmune diseases in dogs are common and complex like human disease, and have the potential to facilitate translational research and serve as a critical bridge between induced models of disease in rodents and clinical trials in humans.[22–25] Like humans, the canine genome has been completely sequenced and annotated, providing a powerful research platform.[26] Dogs and humans have co-evolved in the last 32,000 years, sharing the same environment and evolutionary stressors, leading to an overlap in numerous positively selected genes in multiple key genetic pathways such as immunity, inflammation, neurological process and cancer.[23,27–29] However, experimental methods to detect and manipulate Th17 pathways and data regarding Th17/IL17involvement in canine idiopathic inflammatory and autoimmune disorders are very limited.[30–34]

Multipotent stromal/stem cell (MSC) therapy for Th17 driven diseases is a promising, novel therapeutic option. MSCs are somatic stem cells that may be harvested, isolated and expanded ex-vivo for therapeutic administration.[35,36] These cells are characterized by a spindle morphology, plastic adherence, a specific cell surface phenotype, and the capacity to tri-lineage differentiate in vitro.[37] MSCs secrete a host of paracrine mediators that have potent immunomodulatory, pro-angiogenic and anti-apoptotic properties and they can recruit and dictate the fate of local stem and progenitor cells in vitro and in vivo.[35,38] In humans and mice, MSCs inhibit Th17 polarization and activation via the secretion of prostaglandin E_2_ (PGE_2_) and the induction of myeloid-derived immune suppressive cells and regulatory T (Treg) cells.[39–43] Due to these attributes, MSC based therapies are in advanced (Phase I through III) clinical trials for the treatment of many idiopathic inflammatory and autoimmune disorders that are Th17 driven.[44]

We hypothesized that 1) Th17 cells are present in the blood of healthy dogs and in tissues from dogs with chronic idiopathic inflammatory disorders and that 2) canine MSCs inhibit Th17 polarization. We developed and validated experimental methodologies to explore Th17 pathways in the dog to specifically direct potential application as therapeutic targets for translational regenerative medicine research. We defined and validated protocols to study Th17 pathways in vitro and in vivo in dogs. We demonstrate that Th17 cells are present in the blood of healthy dogs and that IL17 producing cells are present in inflamed tissues from dogs with various chronic idiopathic inflammatory disorders including inflammatory bowel disease, ginigivitis, chronic idiopathic rhinitis and chronic dermatoses. Finally, we show that, like human and murine MSCs, canine MSCs inhibit Th17 polarization in vitro.

## Materials and Methods

### Animal use and cell culture

The protocols for this study were approved by the Institutional Animal Care and Use Committee and the Clinical Trials Review Board at the University of California, Davis (UCD). Blood was collected via jugular venipuncture directly into 10ml heparinized vacutainer tubes (Becton, Dickinson and Company (BD), Franklin Lakes, NJ). Blood was collected from healthy dogs that serve as blood donors at UCD, William R. Pritchard Veterinary Medical Teaching Hospital blood bank. All dog owners signed an informed consent form. Cryopreserved canine fat-derived MSCs were cultured and expanded exactly as previously described.[45] All experiments were conducted using MSCs at passages 3–6. Madin-Darby Canine Kidney (MDCK) Epithelial cells were obtained from American Type Culture Collection.

### Blood CD4+ T cell isolation

Peripheral blood mononuclear cell (PBMC) isolation was carried out via a differential centrifugation method exactly as described by Kol et al.[45] PBMCs were harvested and enumerated using an automated cell counter (Coulter ACT diff, Beckman Coulter, Brea, CA). PBMCs were depleted of monocytes, B cells and granulocytes via an LD column (MACS Separation Columns, Miltenyi Biotec, Auburn, CA) using a cocktail of mouse anti-canine antibodies including anti-CD11b (clone CA16.3E10), anti-CD8α (clone CA9.3D3), anti-CD21 (clone CA2.1D6) and goat-anti mouse IgG-microbeads (Miltenyi Biotec) according to the manufacturer’s instructions. Flow through cells were collected and treated with an anti-canine CD4 antibody (clone CA13.1E4) followed by goat-anti mouse IgG-microbeads (Miltenyi Biotec) and run on an MS column (MACS Separation Columns, Miltenyi Biotec), according to the manufacturer’s instructions, to yield the final CD4+ T cell enriched fraction. All mouse anti-canine antibodies were purchased from the Leukocytes Antigen Biology Lab, UCD School of Veterinary Medicine.

### MSC/CD4+ T cell co-culture

For MSC and CD4 co-culture experiments, MSC and CD4 T cells were plated in a 1:5 ratio and Th17 polarization was induced as described below. Indomethacin (Sigma-Aldrich, St. Louis, MO), a cyclooxygenase (COX) inhibitor, was used to chemically block PGE_2_ production. Indomethacin was added to MSC-CD4 T cell co-culture assays during plating at a concentration of 10μM exactly as previously described.^43^

### Induction of Th17 polarization

Isolated T cells were resuspended in complete lymphocyte media as previously published.[46] Th17 polarization was induced in 2 steps: T cells were activated with 5 μg/ml Concanavalin A (Con A, Sigma-Aldrich) and Th17 polarization was induced via a cytokine cocktail that included 10 ng/ml recombinant canine IL1β (Kingfisher Biotech, St Paul, MN), 5 ng/ml recombinant canine IL6 (Kingfisher Biotech), 2 ng/ml recombinant canine TGF-β (Kingfisher Biotech) and 2 ng/ml neutralizing anti-canine IL4 antibody (clone 140429, R&D systems, Minneapolis, MN). Cells were further cultured in standard conditions (humidified incubator, 37°C, 5% CO_2_) prior to any further analysis.

### Flow cytometry

The protocol for cytokine production and intracellular accumulation for flow cytometric detection was adopted from C. L. Fellman et al. and modified.[46] Cells were treated with 25 ng/ml Phorbol-12-Myristate-13-Acetate (PMA, Sigma-Aldrich) and 500 ng/ml ionomycin (Sigma-Aldrich). After 3 hours of incubation, 1 μg/ml Brefeldin-A (Sigma-Aldrich) was added and cells were cultured for additional 3 hours. Cells were then washed, stained with a viability dye (Fixable Viability Dye eFlour^®^780, eBioscience), fixed and permeabilized (Foxp3/Transcription factor fixation/permeabilization concentrate and diluent, eBioscience) and stained with the following primary conjugated antibodies: anti-canine CD3-AlexaFluor488 (clone CA17.2A12, Leukocyte antigen biology lab, UCD), anti-canine CD4-PE (CA13.1E4, Leukocyte antigen biology lab, UCD) and anti-human IL17-AlexaFluor647 (Goat anti-human IL17, R&D systems). Anti-IL17 antibody was conjugated using Alexa Fluor 647 monoclonal antibody labeling kit (Invitrogen, Carlsbad, CA) per manufacturer’s instructions. Fluorescence was detected by a flow cytometer (Cytomics FC500, Beckman Coulter) and flow cytometry data were analyzed using FlowJo flow cytometry software (Tree Star Inc.).

### Immunobloting

To confirm the specificity of the anti-human IL17 antibody that was used, a western blot analysis of supernatants was performed. Briefly, supernatant from polarized and resting CD4+ T cells was spun twice (400g x 10 minutes, followed by 2500g x 5 minutes) to remove cellular elements, boiled (95°C x 3 minutes), loaded into a 4–20% SDS Precast Gel (Expedeon, San Diego, CA) and separated by electrophoresis. Proteins were transferred to PVDF membranes overnight and then probed overnight with the same anti-human IL17 antibody that was used for flow cytometric detection. Membranes were washed, followed by 1 hour incubation with HRP conjugated-rabbit anti-cat IgG antibody, and finally incubated for 1 minute with Pierce ECL Western Blotting Substrate (Thermo Scientific, Pittsburgh, PA). Digital images were obtained via a FlourChem E imaging system (ProteinSimple, San Jose, CA).

### IL17 ELISA

Media was aspirated and cells were spun down. Supernatant was aspirated off and IL17A concentration was determined with an anti-canine IL17 antibody by DuoSet ELISA (R&D systems) per manufacturer’s instructions.

### Gene expression

T helper cells were washed twice with Dulbecco’s Phosphate Buffered Saline (DPBS), lysed with RLT buffer and RNA was extracted (RNAeasy mini kit Qiagen, Gaithersburg, MD) per manufacturer’s instructions. cDNA was synthesized (First-Strand cDNA synthesis Origene, Rockville, MD) per manufacturer’s instructions. Quantitative PCR (qPCR) was performed on a 7300 Real Time PCR System (Applied BioSystems, Foster City, CA). Primers were designed using Integrated DNA technology website (http://www.idtdna.com/Primerquest/Home/Index) with sequences from Genebank accession numbers (Table 1). Changes in gene expression were calculated by the ΔΔCT method[47] and depicted as fold change in gene expression compared to control.

**Table 1:** Primers sets used for the qPCR assays.

Target	Forward primer sequence	Forward primer sequence	Product size (bp)
Canine RORa	GGCTTCTTTCCCTACTGTTCTT	CAGAATATATCTAAATCACATCTG	112
Canine RORc	CTTACAATGCTGACAACCACAC	CATCTTTGACTTCTCCCGCT	114
Canine IL17A	CAATGAGGACCCTGAGAGATAC	GACGGAGTTCATGTGGTAGTT	106
Canine IL17F	AGTGTGAGGGTTGACATTCG	GTCGCGGGTAATGTTGTAGT	108
Canine CCR6	TGTCCTCACTCTCCCATTCT	AGTTGAAGTTGATGGCGTAGAT	106
Canine IL23R	CACAGACTACAAGGCGGAAA	TTGTGTATATTCCTGGTCTCAGC	106

To confirm the specificity of our primers, qPCR products were run on a 2% agarose gel and product size as well as the presence of non-specific products were determined. IL17A and RORc qPCR products were further cloned into 2.1 PCR product vector per manufacturer’s instructions (TOPO-TA Cloning Kit, Life technologies). Plasmids were then transfected into OneShot^®^ cells and positive colonies selected and expanded overnight prior to plasmid isolation (Wizard^®^ Plus Minipreps DNA Purification System, Promega, Madison, WI) and sequencing by an automated sequencer (Nucleics, Davis, CA).

### Immunofluorescence

Archived formalin fixed and paraffin embedded (FFPE) tissues were identified by searching the Veterinary Medical & Administrative Computer System at UCD School of Veterinary Medicine. Five μm tissue sections were cut and mounted onto glass slides. Tissues were deparaffinized with xylene and a serial ethanol dilution. Slides were boiled at 95°C for 20 minutes (Target Retrieval Solution, DAKO, Carpinteria, CA) following blocking with 15% donkey serum (Jackson ImmunoResearch Laboratories Inc, West Grove, PA) and 1% Fc blocker (Miltenyi Biotec) for 45 minutes. Slides were than incubated with rabbit anti-human CD3 (DAKO) and goat anti-human IL17 (R&D) antibodies at 4°C overnight. Slides were then washed and stained with donkey anti-rabbit AF488 (Invitrogen) and donkey anti-goat AF555 (Invitrogen) for 1 hour at room temperature in the dark. Finally, slides were mounted with DAPI containing mounting media (Vector Laboratories, Burlingame, CA). Images were acquired with a LSM 700 confocal microscope (Zeiss, Pleasanton, CA).

### Statistical analysis

Normal distribution of the data was tested using the Shapiro-Wilk test. A paired t test (normally distributed data) or Mann–Whitney U test (non-normally distributed data) was used to determine differences in flow-cytometric, gene expression and protein secretion data. A commercially available statistical software was used for all statistical analyses (GraphPad InStat version 3.06 for Windows; GraphPad, La Jolla, CA, USA). A P value of <0.05 was considered statistically significant.

## Results

### Canine Th17 cells can be quantified in peripheral blood via flow cytometry

**Fig 1:**
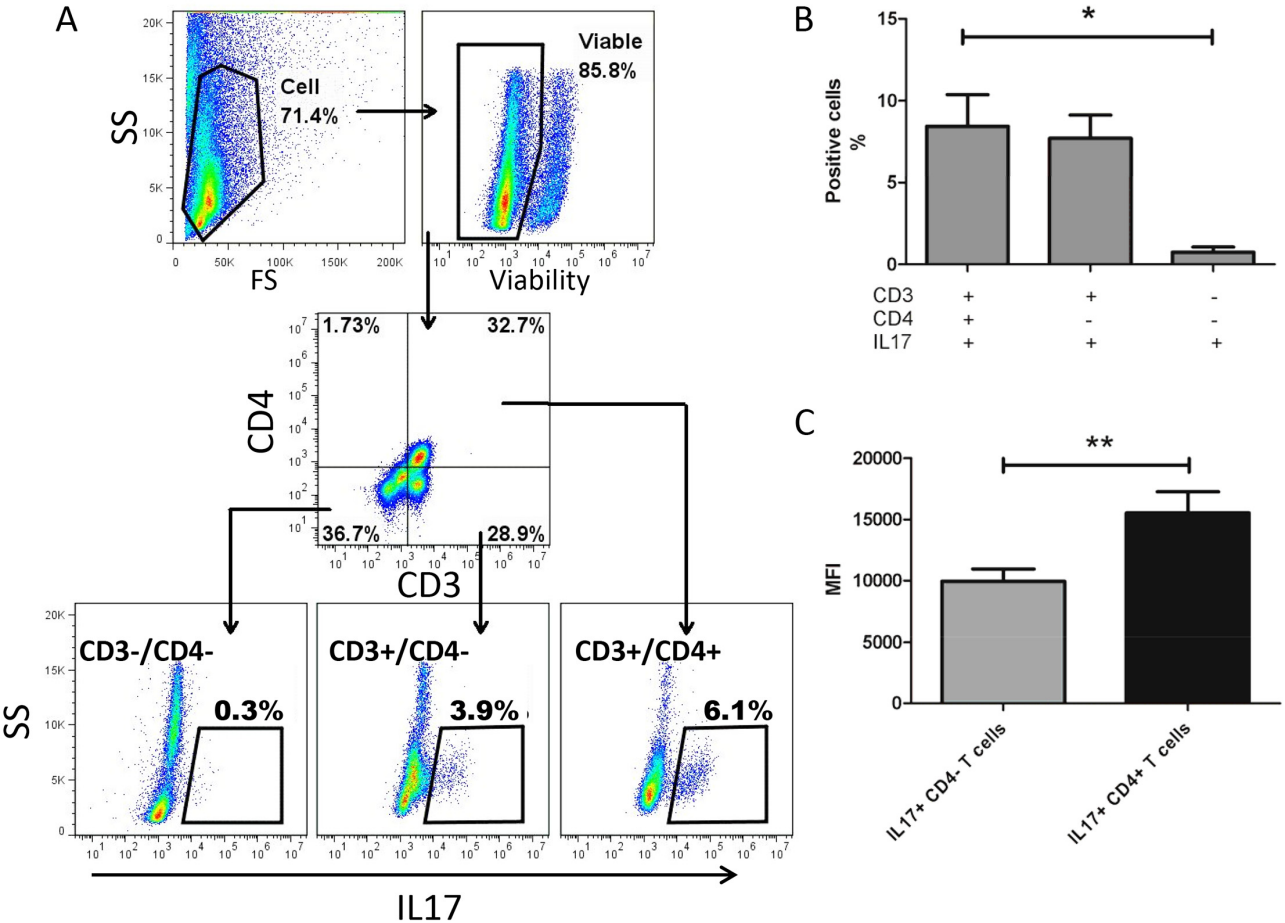
Th17 detection strategy and baseline levels in healthy dogs. Panel A depicts the gating strategy that was employed to detect circulating Th17 cells in dogs. Peripheral blood mononuclear cells were isolated, stimulated with PMA and ionomycin and stained with CD3, CD4, IL17 and a viability dye as described in the methods. Viable cells were gated and intracellular IL17 was detected within Th cells (i.e. CD3+/CD4+), non-Th cells (primarily CD8 T cells, CD3+/CD4-) and within non T cells (primarily B cells, CD3-). The percentages of the various lymphocyte subsets that were IL17 positive (B) and the MFI of IL17 within Th17 cells (i.e. CD3+/CD4+) as compared with CD4- T cells (C) are depicted. Note that while CD4+ and CD4- T cells had similar proportion of IL17 positive cells (B), CD4+ T cells had higher MFI compared with CD4- T cells (C), suggesting greater capacity to produce IL17 upon stimulation. Data are represented as mean +/- standard deviation, n = 5. (* P<0.05, ** P<0.01)

To facilitate flow-cytometric detection of Th17 cells in peripheral blood, PBMCs were isolated from whole blood and cytokine production and accumulation was initiated by stimulating the cells with PMA, ionomycin and brefeldin-A. Fig 1A depicts the gating strategy that was employed to define cell populations. IL17 producing cells accounted for 8.4% +/- 4.3% of CD4+ T cells (i.e. Th17 cells) in canine blood (mean +/- standard deviation (SD), n = 5, Fig 1B). Almost equal numbers of IL17 producing T cells were CD4- (7.7% +/- 3.2%). CD4+ Th17 cells had a significantly higher (P<0.01) IL17 mean fluorescence intensity (MFI) than CD4- T cells, suggesting that a greater amount of IL17 was produced by these specialized Th17 cells (Fig 1C). Only rare (0.7% +/- 0.7) CD3- cells produce IL17 upon in vitro stimulation. The percentage of IL17+ cells within the CD3- cells was significantly lower (P<0.05) than in the CD4+ T cells. In order to determine the specificity of the polyclonal anti-human IL17 antibody that was used, canine IL17 containing media was electrophorated and probed with the same antibody (S1 Fig). Western blot analysis indicated that 2 protein bands were detected with the appropriate molecular weight (~17 and 20 kDa), consistent with native and glycosylated canine IL17.[9] These findings confirmed that the polyclonal anti-human IL17 antibody was specific for canine IL17.

### IL17 producing cells are found in tissues affected by chronic idiopathic inflammation in dogs

**Fig 2:**
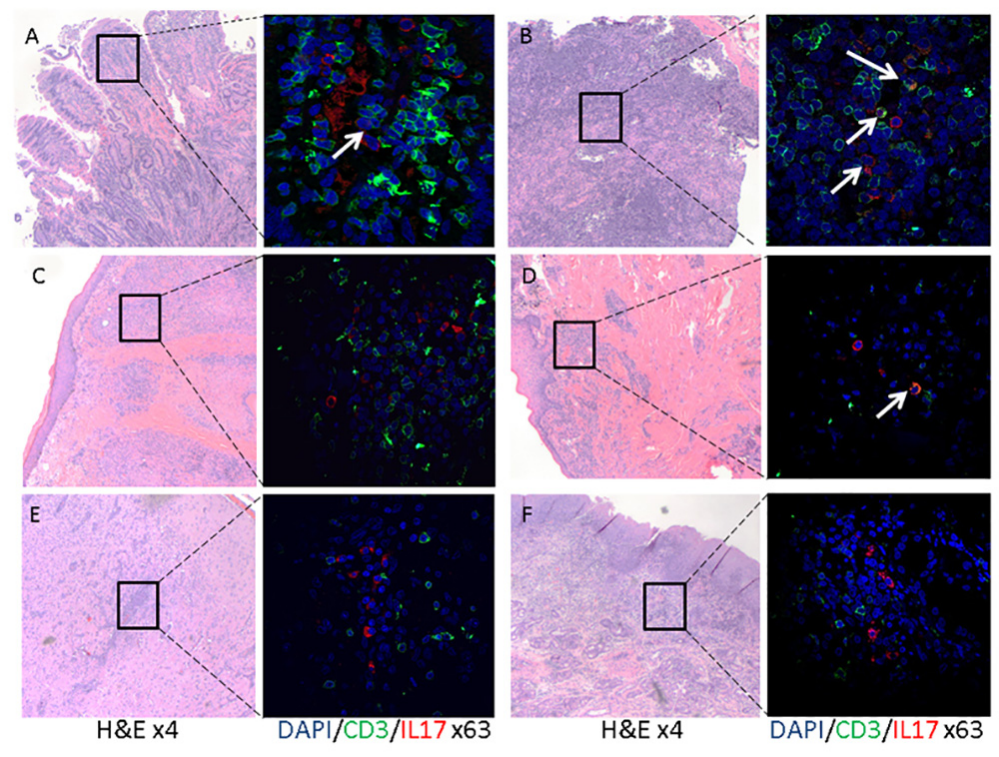
IL17 and CD3 dual detection in idiopathic chronic inflammatory lesion in dogs. FFPE tissues from dogs with naturally occurring diseases were identified, stained and scanned as described in the Material and Methods section. Each panel (A-F) consists of a low power (objective X4) image of an H&E stained slide and a high power (objective X63) image of the same slide stained with CD3 (green), IL17 (red) and DAPI (blue). Presented are representative images from the duodenum (A) and mesenteric lymph node (B) of dogs (n = 5) with inflammatory bowel disease, skin (C) of a dog with chronic idiopathic dermatitis, gingiva (D) of dogs (n = 5) with chronic idiopathic gingivitis, cerebrum (E) of a dog (n = 1) with necrotizing meningoencephalitis and nasal mucosa (E) of dogs (n = 3) with chronic rhinitis. CD3 positive (i.e. green) and IL17 positive (i.e. red) cells are present in all of the represented lesions. Low numbers of double positive cells (indicated by white arrows) are present while the majority of the IL17 positive cells are CD3 negative.

After the successful identification of Th17 cells via flow cytometry in healthy dog blood, we next investigated whether IL17 producing T cells were present in chronic idiopathic inflammatory lesions in dogs. We selected lesions that had histopathologic evidence of chronic lymphocytic-neutrophilic-histiocytic inflammation, where no primary etiology could be identified (Fig 2). IL17 positive cells were detected in duodenal and mesenteric lymph node tissues from dogs with inflammatory bowel disease (Fig 2A and 2B, n = 5), chronic dermatoses (Fig 2C, n = 1), chronic gingivitis (Fig 2D, n = 5), necrotizing meningoencephalitis (Fig 2E, n = 1), and chronic lymphoplasmacytic and neutrophilic rhinitis (Fig 2F, n = 3). Overall, IL17 positive cells were a rare population with the highest number of cells noted in the inflamed intestinal mucosa of dogs with inflammatory bowel disease and in gingival tissues of dogs with chronic gingivitis. The majority of IL17+ cells were CD3- and only <10% were double positive (Fig 2, white arrows). These findings indicate that IL17 producing immune cells are present and are easy to identify in many tissue types in naturally occurring chronic idiopathic inflammatory disorders in dogs. These findings set the stage for determining the role of IL17 producing cells in a wide array of chronic idiopathic inflammatory lesions in the dog.

### Blood derived canine CD4+ T cells can be efficiently polarized into Th17 cells in vitro

**Fig 3:**
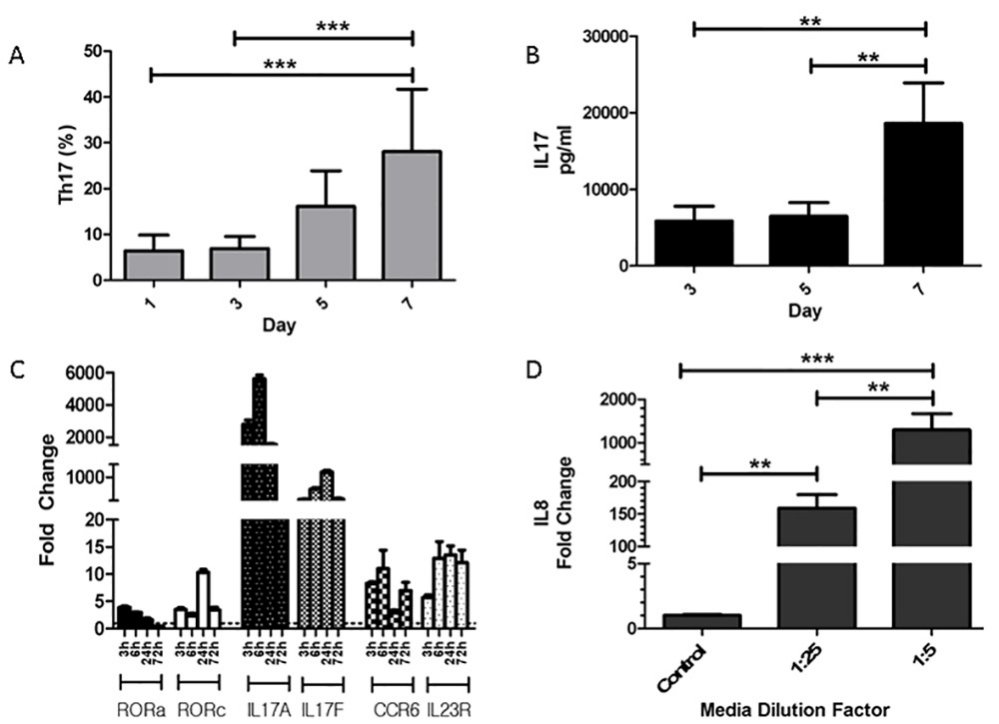
Induction of Th17 polarization in canine Th cells in vitro. Peripheral blood derived CD4+ T cells from healthy dogs (n = 7) were isolated and stimulated as described in the Material and Methods section. Th17 polarization was determined by flow cytometric detection of intracellular IL17 (A), secreted IL17 via ELISA (B) and gene transcription via qRT-PCR (C). Th17 detection by flow cytometry was strongly correlated with ELISA detection of secreted IL17 and both had reached a maximum at 7 days post stimulation (P<0.001). Quantification of key transcription factors (RORa and RORc), cytokines (IL17A and IL17F) and cell surface markers (CCR6 and IL23R) were markedly upregulated during the first 72 hours post stimulation and had picked at 24 hours post induction of polarization. Media from polarized cultures induced dose-dependent gene expression of IL8 in canine epithelial cells (i.e. MDCK cells (D). (** P<0.01, *** P<0.001)

In order to establish an in vitro platform to study Th17 polarization and activation in the dog, we established an in vitro Th17 polarization protocol for blood derived canine CD4+ T cells. Peripheral blood derived CD4+ T cells were activated in the presence of a polarizing cytokine cocktail. The kinetics of Th17 polarization was determined via Th17 cell identification, secreted IL17 protein and transcription of key genes in the Th17 pathway (Fig 3). We found that maximal Th17 polarization was achieved at day 7 post initiation of polarization. At day 7, Th17% was 28% +/-13.6 (n = 7, Mean +/- SD, Fig 3A) and IL17 concentration in the supernatant was 18,585 pg/ml +/- 14,051 (n = 7, Mean +/- SD, Fig 3B). Key genes in the Th17 pathway, including the master transcriptional regulators, RORa and RORc, cytokines (IL17A and IL17F) and cell surface receptors (CCR6 and IL23R), were all statistically significantly upregulated during the polarization protocol (Fig 3C). The transcription of RORc was upregulated primarily within the first 24 hours, with a ∼10 fold increase. Gene transcription of the IL17 family member cytokines were upregulated up to several thousand fold increase. In order to determine that the secreted IL17 was biologically active, we applied conditioned media (i.e. media supernatant from day 7 cultures) onto cultured canine epithelial cells (MDCK cells) and determined its capacity to induce transcription of the key neutrophil chemotactic factor IL8 (aka CXCL8). Media that contained high concentrations of secreted IL17 induced significant upregulation of IL8 transcription in canine epithelial cells in a dose dependent fashion (Fig 3D).

### Canine MSCs inhibit Th17 polarization in vitro in a PGE_2_ independent fashion

**Fig 4:**
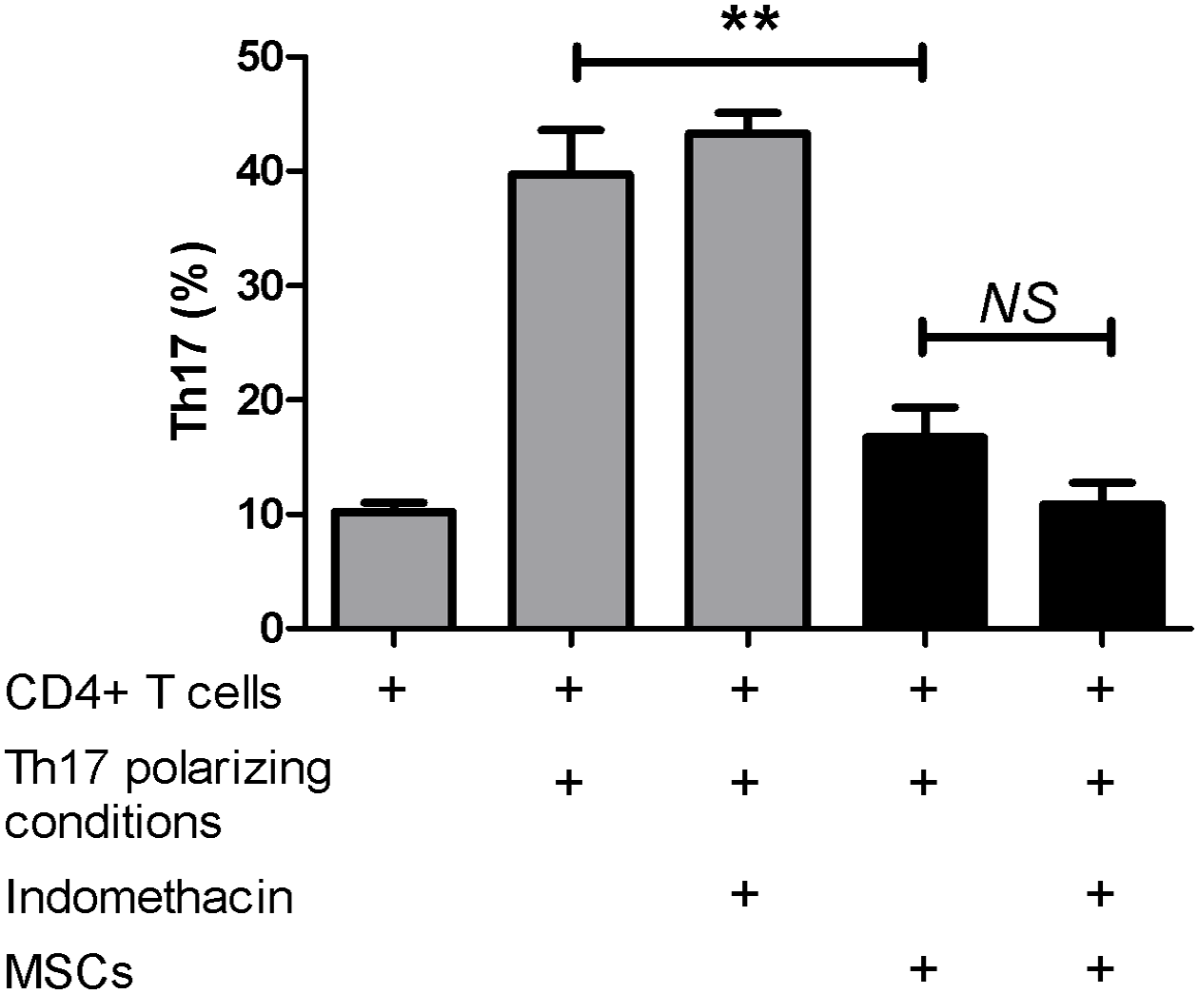
MSC inhibit Th17 polarization in vitro in a PGE_2_ independent fashion. Canine CD4+ T cells (n = 3) were co-incubated with fat derived canine MSCs (n = 7) during the Th17 polarization protocol. Indomethacin, a COX inhibitor was added to some wells to determine the role of MSC-derived PGE_2_ in mediating the MSCs’ inhibitory effect. The addition of indomethacin to the CD4+ T cells that were stimulated in the absence of MSCs did not alter Th17 polarization. Co-culture of stimulated CD4+ T cells in the presence of MSCs induced marked and significant (P<0.05) inhibition of Th17 polarization. Blocking PGE_2_ did not rescue Th17 polarization, suggesting that PGE_2_ does not play an inhibitory effect in canine Th17 polarization. This experiment was repeated three times.

Finally, as our group is focused on the development and translation of novel MSC-based therapeutics to treat inflammatory disorders, we wanted to see if canine MSCs inhibit Th17 polarization in vitro. As PGE_2_ has been implicated in the mechanism by which MSCs inhibit Th17 polarization in human CD4+ T cells, we investigated whether PGE_2_ secretion by canine MSCs plays a similar role in the inhibition of canine Th17 polarization. Th17 polarization was induced in CD4+ T cells in the presence or absence of MSCs with or without the COX inhibitor, indomethacin (Fig 4). The experiment was carried out with T cells from 3 donors and a total of 7 MSC lines. While Th17 polarization yield 39.7% +/- 6.7 (mean +/- SD) Th17 cells with our Th17 polarization protocol, Th17 polarization was significantly inhibited (P<0.01) in the presence of MSCs yielding 16.7% +/- 8.6 Th17 cells (mean +/- SD). When PGE_2_ secretion was inhibited with indomethacin, Th17 polarization did not change. These findings suggest that canine MSC inhibit Th17 polarization in vitro in a PGE_2_ independent fashion.

## Discussion

This is the first paper to identify Th17 cells in peripheral blood and IL17 producing immune cells in inflamed tissues in healthy dogs and dogs with naturally occurring chronic inflammatory diseases, respectively. We define Th17 polarization kinetics in dogs by protein production and gene transcription that will enable further mechanistic study of this pathway and permit the development of novel approaches to target Th17 polarization in vitro. Similar to humans and mice, canine MSCs inhibit Th17 polarization in vitro, suggesting that naturally occurring diseases in dogs may serve as good models to translate novel MSC-based therapeutics for IL17 driven diseases.

IL17 driven autoimmune/autoinflammatory diseases represent a group of severe immune disorders in which the development and FDA approval of novel therapeutic agents is lagging behind the advances made in pre-clinical studies in induced models of disease in laboratory animals. There is increasing appreciation in the wider biomedical community that the study of naturally occurring disease in dogs may serve as an excellent platform to conduct translational research.[21] Naturally occurring diseases in dogs share some of the complexities of human diseases such as heterogeneous genetic background, environmental interactions, longevity and the availability of a model heath care system–advanced veterinary medicine.[21]

In peripheral blood, IL17 was detected both in CD4+ T cells (aka Th17) as well as in CD4- T cells, which most likely represent CD8+ cells (Tc17).[48] Interestingly, IL17 MFI was significantly higher in Th17 cells (CD4+) compared with CD4- T cells, suggesting that traditional Th17 cells are able to produce higher concentrations of IL17 upon stimulation. As expected, IL17 was not produced by non T cells in peripheral blood. Although we did not directly compare the proportions of circulating dog and human Th17 cells, it appears that dogs may have higher numbers of circulating Th17 cells in health.[49–52]

Unlike blood, where T cells are the primary source of IL17, in all of the inflamed tissues, IL17 was mostly present in CD3- cells. The finding of IL17 producing non-T-cells in tissue sections is consistent with a previous report of inflammatory brain lesions in dogs, where most of the IL17+ cells were CD3-.[32] This has also been described in human patients with oral inflammation,[53] chronic lymphocytic leukemia[54] and in normal[55] and post *Salmonella typhimurium* infection of non-human primate intestinal tissues (Satya Dandekar, unpublished data). Nevertheless, CD4+/IL17+ or CD3+/IL17+ double positive cells have been described in inflamed sites including the intestinal tract,[1] skin,[56] brain[57] and synovial membrane.[4,58] The discrepancies between blood and tissues may be partially explained by the different detection methodologies. In flow cytometry it is common to stimulate the cells with PMA and ionomycin prior to analysis whereas immunofluorescent analysis of tissue sections is conducted on fixed, and otherwise untreated cells. These findings may suggest that IL17 production has to be stimulated in CD4+ Th17 cells prior to detection and that CD3-/IL17+ cells may represent a constitutively active, IL17 secreting immune cell subtype such as innate lymphoid cells, macrophages or others.[10] Further characterization of these unusual CD3-/IL17+ cells is warranted.

Our in vitro Th17 polarization assay confirmed that, like human Th17 cell polarization, Th17 polarization in the dog is induced by T cell activation in the context of the key cytokines TGF-β, IL6 and IL1β.[59,60] We also confirmed that the secreted IL17 had biological activity by demonstrating its induction of IL8 gene expression in a canine epithelial cell culture. This canine specific in vitro polarization assay may be utilized to study novel therapeutic approaches that target the Th17 polarization cascade and to illuminate species specific differences in this pathway. In this study, due to limitations in reagent availability, the entire CD4 T cell fraction was isolated which includes not only naïve Th cells, but also central and effector CD4+ T memory cells. This limitation prohibited us from discriminating between de novo Th17 differentiation of naïve CD4 T cells versus the expansion of memory Th17 cells.[61]

Finally, our data demonstrate that like human and murine MSCs, canine MSCs have the capacity to inhibit Th17 polarization in vitro. Nonetheless, while PGE_2_ secretion by MSCs was implicated as a critical factor that mediates human MSC inhibition of Th17 polarization, secretion of PGE_2_ by canine MSCs does not seem to play a significant role in the inhibition of canine Th17 polarization.[41,42] These findings are consistent with previous publications from our laboratory indicating that while canine MSCs secrete abundant PGE_2_, it does not play a significant role in the inhibition of canine lymphocyte proliferation by MSCs.[45,62] PGE_2_ has also been implicated in various experimental settings as an enhancer of Th17 polarization and activation.[63,64] These data suggest that lymphocyte proliferation and Th17 differentiation may be regulated by canine MSCs, although the exact mechanism and the role of PGE_2_ need to be further defined. Ultimately, our results warrant further investigation into the potential role that canine MSCs may have in inhibiting Th17 polarization in vivo. As such, Th17 driven disease in dogs may be valuable models for novel MSC-based translational research.

This work provides fundamental research tools and sets the foundation for future research of IL17 and Th17 driven diseases in dogs and into the potential ways to target these disease with MSC-based novel therapeutics. It further emphasizes the great translational potential of naturally occurring chronic inflammatory diseases in dogs.

## Supporting Information

S1 Fig Goat anti-human IL17 antibody and qPCR primer validation. Given the lack of canine specific IL17 antibodies for flow cytometry and lack of validated canine qPCR primers, we used a polyclonal goat anti-human IL17 antibody and self-designed qPCR primers. Validation of the anti-IL17 antibody included the analysis of flow cytometry data which indicated that the antibody recognizes an antigen that is expressed in the appropriate cell type (i.e. T cells and not in non-T cells) and in the expected proportion of positive cells in healthy dogs. We confirmed by western blot analysis that the antigen that is being detected by the antibody has the appropriate size bands(~16 and 20 kDa, A). The bands are likely to represent backbone (i.e. the ~16 kDa band) and N-linked glycosylated form of the IL17 (i.e. the ~20 kDa band). The specificity of our self-designed qPCR primers was verified by electrophoresing the PCR products in a 2% agarose gel and determining product size, the presence of additional PCR products and the presence of primer dimers (B). All PCR products had the expected size and no additional products or primer dimers were detected. We further confirmed RORa and IL17A PCR products by DNA sequencing. Sequence analysis confirmed that the sequences are 100% identical with canine RORa and IL17A mRNA sequences (C).
(TIF)
