# Supplementary figures and images for: Th17 Pathway As a Target for Multipotent Stromal Cell Therapy in Dogs: Implications for Translational Research
Source: PLoS One. 2016 Feb 12;11(2):e0148568. doi: 10.1371/journal.pone.0148568 (PMC4752288; doi:10.1371/journal.pone.0148568)

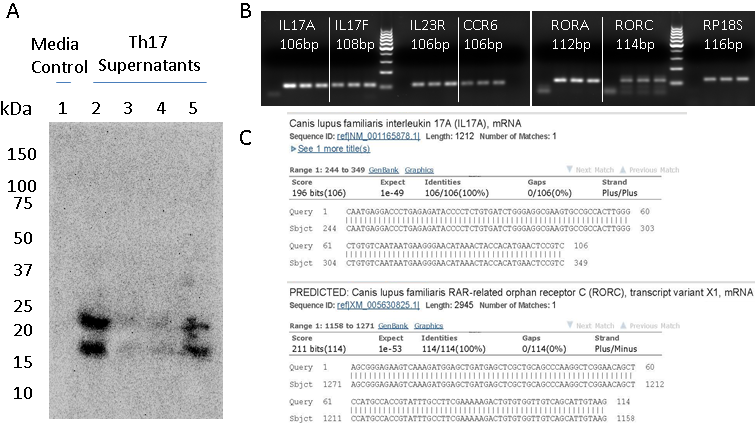

Supplement: S1 Fig — Given the lack of canine specific IL17 antibodies for flow cytometry and lack of validated canine qPCR primers, we used a polyclonal goat anti-human IL17 antibody and self-designed qPCR primers. Validation of the anti-IL17 antibody included the analysis of flow cytometry data which indicated that the antibody recognizes an antigen that is expressed in the appropriate cell type (i.e. T cells and not in non-T cells) and in the expected proportion of positive cells in healthy dogs. We confirmed by western blot analysis that the antigen that is being detected by the antibody has the appropriate size bands(~16 and 20 kDa, A). The bands are likely to represent backbone (i.e. the ~16 kDa band) and N-linked glycosylated form of the IL17 (i.e. the ~20 kDa band). The specificity of our self-designed qPCR primers was verified by electrophoresing the PCR products in a 2% agarose gel and determining product size, the presence of additional PCR products and the presence of primer dimers (B). All PCR products had the expected size and no additional products or primer dimers were detected. We further confirmed RORa and IL17A PCR products by DNA sequencing. Sequence analysis confirmed that the sequences are 100% identical with canine RORa and IL17A mRNA sequences (C). (TIF) [file pone.0148568.s001.tif]
